# Draft Genome Sequence of *Streptococcus agalactiae* Serotype Ia Strain M19, a Multidrug-Resistant Isolate from a Cow with Bovine Mastitis

**DOI:** 10.1128/genomeA.01093-16

**Published:** 2016-11-03

**Authors:** Feng Yang, Hongsheng Li, Shidong Zhang, Xurong Wang

**Affiliations:** Lanzhou Institute of Husbandry and Pharmaceutical Sciences of Chinese Academy of Agricultural Sciences, Lanzhou, Gansu, People's Republic of China

## Abstract

*Streptococcus agalactiae* is a major contagious pathogen causing bovine mastitis worldwide. We report here the draft sequence of *S. agalactiae* Ia strain M19, a multidrug-resistant isolate from a bovine mastitis case in Ningxia Hui autonomous region, China.

## GENOME ANNOUNCEMENT

*Streptococcus agalactiae* is a major contagious pathogen causing bovine mastitis, which may have a substantial impact on the quantity and quality of produced milk (1). This bacterium is classified into 10 serotypes based on the antigenic diversity in the capsular polysaccharide (2). Strains of serotypes Ia and II are the most commonly encountered in bovine mastitis in China (data not shown). Here, we report the draft genome sequence of *S. agalactiae* Ia strain M19, a multidrug-resistant isolate from a cow with bovine mastitis in Ningxia Hui autonomous region, China.

The draft genome sequence of *S. agalactiae* strain M19 was performed using Illumina HiSeq 2000 sequencing platform at the Beijing Genomics Institute (BGI) (Shenzhen, China). Draft assemblies were based on 382-Mb clean data. All reads provided about 177-fold coverage of the genome. The paired-end reads were assembled into four contigs in three scaffolds with the SOAPdenovo program (3, 4). Putative open reading frames were predicted using Glimmer (5). Tandem repeats were selected using Tandem Repeat Finder (6). tRNAs and rRNAs were identified using tRNAscan-SE (7) and RNAmmer (8), respectively. The functions of encoding genes were annotated by using the NCBI nr, Kyoto Encyclopedia of Genes and Genomes (KEGG), and Cluster of Orthologous Groups of proteins (COG), as well as the Antibiotic Resistance Genes Database (ARDB).

The circular chromosome of *S. agalactiae* M19 is composed of 2,111,437 nucleotides with an overall G+C content of 35.61%. It contains 2,056 coding sequences (CDSs) that account for 88.45% of the genome, 1,707 of which (83.0%) were annotatable with known proteins with biological function or a functional domain and 349 of which (17.0%) were annotated as conserved hypothetical proteins. The genome also harbors 68 tandem repeat sequences, including loci for 34 minisatellite DNAs and 3 microsatellite DNAs, as well as 57 tRNAs and 15 rRNAs. ARDB annotated 11 genes conferring resistance to chloramphenicol (one), vancomycin (four), bacitracin (three), tetracycline (one), penicillin (one), and multidrug resistance to ciprofloxacin and norfloxacin (one).

### Accession number(s).

This whole-genome shotgun project has been deposited at DDBJ/ENA/GenBank under the accession number LKPA01000000 with the scaffold accession number KV757177.

## References

[1] Radtke A, Bruheim T, Afset JE, Bergh K 2012 Multiple-locus variant-repeat assay (MLVA) is a useful tool for molecular epidemiologic analysis of *Streptococcus agalactiae* strains causing bovine mastitis. Vet Microbiol 157:398–404. doi:10.1016/j.vetmic.2011.12.034. 22266162

[2] Imperi M, Pataracchia M, Alfarone G, Baldassarri L, Orefici G, Creti R 2010 A multiplex PCR assay for the direct identification of the capsular type (Ia to IX) of *Streptococcus agalactiae*. J Microbiol Methods 80:212–214. doi:10.1016/j.mimet.2009.11.010. 19958797

[3] Li R, Zhu H, Ruan J, Qian W, Fang X, Shi Z, Li Y, Li S, Shan G, Kristiansen K, Li S, Yang H, Wang J, Wang J 2010 De novo assembly of human genomes with massively parallel short read sequencing. Genome Res 20:265–272. doi:10.1101/gr.097261.109. 20019144 PMC2813482

[4] Li R, Li Y, Kristiansen K, Wang J 2008 SOAP: short oligonucleotide alignment program. Bioinformatics 24:713–714. doi:10.1093/bioinformatics/btn025. 18227114

[5] Delcher AL, Bratke KA, Powers EC, Salzberg SL 2007 Identifying bacterial genes and endosymbiont DNA with glimmer. Bioinformatics 23:673–679. doi:10.1016/10.1093/bioinformatics/btm009. 17237039 PMC2387122

[6] Benson G 1999 Tandem repeats finder: a program to analyze DNA sequences. Nucleic Acids Res 27:573–580. doi:10.1093/nar/27.2.573. 9862982 PMC148217

[7] Lowe TM, Eddy SR 1997 tRNAscan-SE: a program for improved detection of transfer RNA genes in genomic sequence. Nucleic Acids Res 25:955–964. doi:10.1016/10.1093/nar/25.5.0955. 9023104 PMC146525

[8] Lagesen K, Hallin P, Rødland EA, Staerfeldt H-H, Rognes T, Ussery DW 2007 RNAmmer: consistent and rapid annotation of ribosomal RNA genes. Nucleic Acids Res 35:3100–3108. doi:10.1016/10.1093/nar/gkm160. 17452365 PMC1888812

